# Supplementary material for: Machine Learning-Assisted Large-Area Preparation of MoS2 Materials
Source: Nanomaterials (Basel). 2023 Aug 9;13(16):2283. doi: 10.3390/nano13162283 (PMC10459608; doi:10.3390/nano13162283)
Supplement: Supplementary file 1 [file nanomaterials-13-02283-s001.zip › nanomaterials-2500667-supplementary.pdf]

Supporting information

Machine Learning Assisted Large Area Preparation of MoS_2 Materials

Jingting Wang, Mingying Lu, Yongxing Chen, Guolin Hao, Bin Liu, Pinghua Tang, Lian Yu, Lei Wen and Haining Ji*

Table S1. Datasets from literatures and laboratories.

No.	R(Mo:S)	Flow rate (sccm)	Raction tem-perture (K)	Raction time(min)	Size(μm)
1	0.020	50	1071.15	25	40.000
2	0.020	50	1071.15	10	10.000
3	0.020	50	1071.15	20	25.000
4	0.020	50	1046.15	10	20.000
5	1.000	25	951.15	10	50.000
6	0.020	25	951.15	5	1.500
7	0.020	50	1071.15	15	20.000
8	0.020	140	921.15	15	7.000
9	0.020	140	971.15	15	16.500
10	0.020	140	1021.15	15	3.000
11	0.020	140	971.15	5	5.000
12	0.020	140	971.15	10	8.000
13	0.020	140	971.15	20	32.000
14	0.040	140	971.15	15	7.400
15	0.050	140	971.15	15	8.900
16	0.060	140	971.15	15	12.600
17	0.070	140	971.15	15	18.400
18	0.090	140	971.15	15	61.000
19	0.15	140	971.15	15	100.000
20	0.100	100	1071.15	25	45.000
21	0.100	30	1071.15	10	50.000
22	0.500	70	921.15	360	15.000
23	0.500	70	921.15	240	5.000
24	0.300	75	1121.15	10	125.000
25	0.333	150	1021.15	10	40.309
26	0.400	100	1121.15	15	54.037
27	0.100	100	1071.15	45	50.313
28	0.333	75	971.15	25	50.000
29	1.000	70	1081.15	20	80.000
30	0.500	120	921.15	180	200.000
31	0.090	50	1091.15	20	20.000
32	0.060	50	1091.15	20	40.000
33	0.060	50	1131.15	20	20.000
34	0.060	50	1171.15	20	28.573
35	0.090	20	1091.15	20	30.000
36	0.015	200	1031.15	20	53.297
37	0.015	200	1031.15	30	60.000
38	0.015	200	1001.15	10	25.000
39	0.013	200	1031.15	10	24.511
40	0.015	200	1031.15	10	23.000
41	0.016	200	1031.15	10	54.627
42	0.028	200	1031.15	10	51.767
43	0.015	100	1031.15	10	46.521

44	0.015	150	1031.15	10	45.179
45	0.015	200	1031.15	10	11.921
46	0.250	20	921.15	60	25.000
47	0.250	20	921.15	40	30.000
48	0.250	20	921.15	20	60.300
49	0.006	60	1101.15	10	47.704
50	0.025	150	1121.15	10	146.000
51	0.020	100	1171.15	20	77.143
52	0.030	100	1171.15	15	161.429
53	0.060	75	1171.15	20	89.048
54	0.030	100	1191.15	20	15.238
55	0.030	100	1171.15	20	167.619
56	0.030	100	1151.15	20	50.476
57	0.100	100	1171.15	20	37.619
58	0.060	100	1171.15	20	42.381
59	0.030	100	1171.15	30	200.000
60	0.250	100	1021.15	5	46.000
61	0.333	100	1021.15	5	15.000
62	0.250	80	1021.15	5	45.000
63	0.004	50	1021.15	10	25.000
64	0.007	50	1021.15	10	6.000
65	0.250	100	1021.15	25	32.000
66	0.250	100	1021.15	28	68.000
67	0.250	100	1021.15	15	7.000
68	0.250	30	1021.15	10	12.000
69	0.250	60	1021.15	40	105.000
70	0.250	100	1021.15	30	72.000
71	0.250	150	1021.15	10	6.000
72	0.100	1000	1121.15	60	29.232
73	0.100	1000	1046.15	60	80.368
74	0.100	1000	898.15	60	65.445
75	0.120	10	906.15	30	72.687
76	0.128	10	906.15	30	31.681
77	0.006	10	906.15	30	30.555
78	0.006	10	1081.15	30	41.939
79	0.006	10	1101.15	30	20.561
80	0.006	10	1121.15	30	16.588
81	0.004	60	1051.15	15	10.341
82	0.004	60	1051.15	20	29.545
83	0.008	100	981.15	10	10.000
84	0.008	100	1001.15	10	40.000
85	0.004	100	1021.15	10	120.769
86	0.008	100	1021.15	10	43.077
87	0.005	100	1021.15	10	300.000
88	0.006	100	1121.15	5	70.000
89	0.006	100	1111.15	5	25.000
90	0.006	100	1101.15	5	30.000
91	0.006	100	1091.15	5	27.549
92	0.006	100	1081.15	5	20.000
93	0.006	100	1071.15	5	15.000
94	0.006	80	1071.15	15	20.000
95	0.006	80	1071.15	20	40.000
96	0.006	80	1071.15	30	89.869
97	0.006	80	1071.15	25	66.177
98	0.667	50	1121.15	15	45.000
99	0.667	50	1021.15	3	7.000
100	0.667	50	1071.15	3	35.000
101	0.667	50	1121.15	30	205.333
102	0.667	50	1171.15	3	90.333

103	0.667	50	1221.15	3	50.000
104	0.020	100	901.15	20	10.000
105	0.020	100	921.15	20	29.310
106	0.020	100	971.15	20	65.086
107	0.020	100	991.15	20	90.000
108	0.100	100	801.15	20	200.000
109	0.100	100	1021.15	20	30.000
110	0.100	100	951.15	20	55.000
111	0.100	100	921.15	15	76.619
112	0.250	100	921.15	15	56.836
113	0.100	35	1001.15	40	13.000
114	0.100	25	1001.15	40	7.000
115	0.100	15	1001.15	40	8.000
116	0.100	5	1001.15	40	15.000
117	0.100	35	1001.15	47	12.500
118	0.100	35	1001.15	52	14.167
119	0.100	35	1001.15	57	25.000
120	0.100	35	1001.15	62	28.750
121	0.015	100	971.15	15	8.000
122	0.018	100	1021.15	15	20.000
123	0.015	100	1071.15	15	60.000
124	0.015	100	1071.15	5	16.204
125	0.015	100	1071.15	30	32.555
126	0.015	50	1071.15	15	22.785
127	0.25	60	1071.15	10	3.000
128	0.150	60	1171.15	10	50.000
129	0.200	60	1171.15	10	63.541
130	0.027	200	1021.15	10	8.333
131	0.027	200	1021.15	15	15.238
132	0.050	70	1271.15	10	10.000
133	0.050	25	971.15	15	80.000
134	0.500	200	1071.15	15	28.667
135	0.500	200	1121.15	15	37.333
136	0.500	200	1171.15	15	24.000
137	0.111	50	1071.15	20	37.931
138	0.111	50	1096.15	20	53.873
139	0.111	50	1121.15	20	40.229
140	0.111	50	1146.15	20	36.782
141	0.111	50	1171.15	20	34.482
142	0.111	75	1071.15	20	81.609
143	0.111	75	1096.15	20	68.956
144	0.111	75	1121.15	20	56.322
145	0.111	75	1146.15	20	45.977
146	0.111	75	1171.15	20	19.154
147	0.111	100	1071.15	20	37.931
148	0.111	100	1096.15	20	72.414
149	0.111	100	1121.15	20	21.893
150	0.111	100	1146.15	20	34.483
151	0.111	100	1171.15	20	18.391
152	0.111	75	1046.15	20	35.263
153	0.500	20	1021.15	5	16.600
154	0.500	20	1071.15	5	15.850
155	0.500	20	1121.15	5	16.900
156	0.500	20	1171.15	5	16.560
157	0.050	100	1071.15	15	20.000
158	0.010	100	971.15	20	7.000
159	0.010	40	971.15	20	8.250
160	0.010	80	971.15	20	20.000
161	0.010	240	971.15	20	20.500

162	0.010	280	971.15	20	9.250
163	0.250	75	951.15	40	8.000
164	0.250	75	971.15	40	35.000
165	0.250	75	1001.15	40	10.000
166	0.250	75	1031.15	40	20.000
167	0.250	75	1061.15	40	13.500
168	0.087	100	921.15	10	0.500
169	0.087	100	971.15	10	1.100
170	0.087	100	1021.15	10	2.000
171	0.087	100	1071.15	10	3.000
172	0.100	100	1071.15	60	50.000
173	0.100	100	991.15	5	80.000
174	0.015	100	921.15	15	10.000
175	0.015	100	1021.15	15	80.000
176	0.015	100	1021.15	5	10.000
177	0.015	100	1021.15	10	40.000
178	0.015	100	1021.15	20	77.000
179	0.015	100	971.15	10	5.000
180	0.015	100	971.15	15	11.316
181	0.015	100	921.15	10	4.298
182	0.015	100	971.15	10	20.000
183	0.015	100	1021.15	30	80.000
184	0.015	100	1071.15	10	40.000
185	0.015	100	1021.15	5	5.000
186	0.015	100	1021.15	10	20.000
187	0.015	100	1021.15	20	40.000
188	0.015	100	1021.15	15	36.934
189	0.015	100	1021.15	25	60.000
190	0.005	100	1021.15	10	20.000
191	0.005	100	1021.15	10	30.000
192	0.015	100	1021.15	15	34.451.
193	0.010	100	1021.15	15	10.000
194	0.005	100	1021.15	15	11.500
195	0.015	100	1021.15	12	27.000
196	0.015	100	1221.15	15	40.000
197	0.010	175	1271.15	15	48.235
198	0.010	150	1271.15	15	45.882
199	0.010	125	1271.15	15	28.353
200	0.010	100	1271.15	15	22.835
